# The N6-Methyladenosine Features of mRNA and Aberrant Expression of m6A Modified Genes in Gastric Cancer and Their Potential Impact on the Risk and Prognosis

**DOI:** 10.3389/fgene.2020.561566

**Published:** 2020-11-04

**Authors:** Liang Sang, Liping Sun, Ang Wang, Han Zhang, Yuan Yuan

**Affiliations:** ^1^Tumor Etiology and Screening Department of Cancer Institute and General Surgery, The First Hospital of China Medical University, Shenyang, China; ^2^Department of Ultrasound, The First Hospital of China Medical University, Shenyang, China; ^3^Key Laboratory of Cancer Etiology and Prevention in Liaoning Education Department, The First Hospital of China Medical University, Shenyang, China; ^4^Key Laboratory of GI Cancer Etiology and Prevention in Liaoning Province, The First Hospital of China Medical University, Shenyang, China

**Keywords:** M6A, methylation, gastric cancer, expression, risk, prognosis

## Abstract

Although N6-methyladenosine (m6A) mRNA methylation is known to be closely related to tumor events, its role in carcinogenesis and the development of gastric cancer (GC) is not yet clear. The aim of this study was to identify common m6A features and novel aberrant expression of m6A modified genes in GC and to further explore their potential impact on risk and prognosis. Three paired GC and paracancerous (PCa) tissues were collected to perform an m6A sequencing by MeRIP-seq and microarray assays. The expression profile of m6A and mRNA were determined. Gene function note and enrichment analysis were performed, and protein–protein interaction networks of differentially m6A methylated genes (DMGs) were generated using the DAVID and STRING databases, respectively. Validation of the m6A related differentially expressed genes by matching TCGA and GTEx data and human tissues. Clinical and pathological correlation and survival analysis were performed by TCGA data. The m6A motif sequence GGACAR (*R* = U or A) C was the consensus in both GC and PCa tissues. m6A peaks were significantly related to different coordinates, however, for most samples, the end of the coding sequence (CDS) was more prominent than the start of CDS. The genes with higher levels of m6A in their mRNAs were mainly enriched in transcriptional misregulation in carcinogenesis pathways, whereas the genes with decreased methylation mainly regulated digestion and absorption of protein. There are genes with differential m6A modifications in GC and paired PCa tissues, and these genes are mainly enriched in transcriptional misregulation and digestion/absorption pathways. m6A-GC with the down- and up-regulated genes may play an important role in gastric carcinogenesis, which can affect the risk and prognosis in GC.

## Introduction

The identification of RNA modifications that regulate gene expression has resulted in the advance of the novel field of “RNA epigenetics” (He, 2010). To date, in multiple RNA species, more than 160 RNA modifications have been identified as post-transcriptional regulatory marks (Boccaletto et al., 2018; Yang et al., 2018). N6-methyladenosine (m6A) is the most prevalent form of mRNA modification in higher eukaryotes, and it plays a significant role in gene expression and metabolism (Wei et al., 1975; Dominissini et al., 2012; Meyer et al., 2012; Huang et al., 2020). m6A was discovered with a canonical RNA motif of RRACH (*R* = A or G; *H* = A, U, or C) and was found to be mainly enriched near stop codons and 3′ untranslated regions (3′ UTRs) (Linder et al., 2015). Because of the involvement of methyltransferases and demethylases, m6A methylation is regulated and reversible in nearly each step of mRNA metabolism, stem cell self-renewal, and cancer progression (Dai et al., 2018; Yang et al., 2018; Chen et al., 2019; Hu et al., 2019; Wang X. et al., 2020).

Recent studies have shown that the methylation of m6A mRNA plays a key role in the occurrence and development of cancers (Lan et al., 2019), such as glioblastoma (Cui et al., 2017; Du et al., 2020), hematologic malignancies (Bansal et al., 2014; Kwok et al., 2017), breast cancer (Zhang et al., 2016a,b), cervical cancer (Wang et al., 2017), hepatocellular carcinoma (HCC) (Ma et al., 2017), renal clear cell carcinoma (Wang J. et al., 2020), and pancreatic cancer (Geng et al., 2020). These studies show that phenotypes could be altered as a result of changes in the expression of key genes related to the function of the m6A modulator. However, for gastric cancer (GC), there are only a few studies on m6A methylation regulators (Xu et al., 2017; Li et al., 2019; Su et al., 2019; Yue et al., 2019). The gene expression effects, the target genes modified with m6A in GC, the extent of such modifications, and the functions and pathways of genes differentially modified with m6A, as well as the potential impacts of the modifications on mRNA expression, all remain elusive.

In this study, we collected three paired GC and paracancerous (PCa) tissues to obtain the first transcriptome-mRNA m6A profiles. Moreover, we investigated the genes with m6A modifications and performed functional annotations and pathway analyses of differentially m6A methylated genes using online tools. Subsequently, we performed a combined data analysis on the genes with differential m6A methylation and expression that were obtained from the sequence and array using GC and PCa tissues, and we explored whether m6A modification could affect mRNA expression, which could be related to the risk and prognosis in GC. Thus, we expected to find common features of m6A and novel aberrantly methylated pathways in GC and key m6A-related genes that might regulate gene expression to play a vital role in gastric carcinogenesis.

## Materials and Methods

### Tissue Specimen Collection

Cancerous tissue and normal tissue (>3 cm adjacent to the cancer) were taken from three surgically removed GC specimens at the First Hospital of China Medical University. Fresh samples were immediately frozen in liquid nitrogen and maintained at −80°C. This study was conducted with the approval of the institutional ethics board of the First Hospital of China Medical University.

### High-Throughput m6A Sequencing

MeRIP-Seq was executed by Cloudseq Biotech Inc. (Shanghai, China) in line with the printed procedure (Meyer et al., 2012) with minor adjustments. In brief, RNA was fragmented and incubated for 2 h with an anti-m6A polyclonal antibody (Synaptic Systems, 202003) in IPP buffer at 4°C. The mixture was then immunoprecipitated by incubation for an extra 2 h at 4°C with protein-A beads (Thermo Fisher). Next, the bound RNA was eluted from the beads with m6A (Berry and Associates, PR3732) in IPP buffer and was then collected with TRIzol reagent (Thermo Fisher) according to the manufacturer’s instructions. The RNA sequence library was generated from purified RNA with a NEBNext^®^ Ultra^TM^ RNA Library Prep Kit (NEB). The m6A IP samples and input samples without immunoprecipitation were both subjected to 150-bp paired-end sequencing on an Illumina HiSeq sequencer.

### mRNA Expression Assay by Microarray

The experiment used NanoDrop ND-2000 (Thermo Scientific) to quantify total RNA and Agilent Bioanalyzer 2100 (Agilent Technologies) to assess RNA integrity. Sample labeling, microarray hybridization, and washing were performed in accordance with the manufacturer’s instructions. In short, total RNA was transcribed into double-stranded cDNA, and then cRNA was manufactured and tagged with cyanine-3-CTP. The tagged cRNAs were hybridized to the microarray. Finally, the array was scanned using an Agilent Scanner G2505C (Agilent Technologies) after washing.

### Data Analysis

#### Analysis of N6-Methyladenosine Features of Differentially Methylated Genes Between GC and Paired PCa Issues

First, quality control was performed by Q30 after obtaining paired-end reads from Illumina HiSeq 4000 sequencer, and then low-quality and 3′ adaptor-trimming reads were deleted by cutadapt software (v1.9.3). Next, HISAT2 software (v2.0.4) was used to align all clean reads of libraries with the reference genome (UCSC HG19). MACS software was used to identify the methylated sites of RNAs (peaks). In addition, diffReps was used to discover differentially methylated sites. In this study, we used *P* < 1e-05 and | fold change| > 2 as the cutoff criteria for differentially methylated genes (DMGs). The conserved m6A motif in GC tissues was constructed by MEME software using 100-nt RNA fragments, including methylation sites.

#### DMGs Related Enrichment Analysis and Protein–Protein Interaction Network Construction

Gene Ontology and pathway enrichment analyses were performed for DMGs using the DAVID database. A protein–protein interaction (PPI) network of genes with increased or decreased m6A levels was built through the STRING (Search Tool for the Retrieval of Interacting Genes/Proteins)^1^ database. An interaction score (median confidence) of 0.4 was adopted as the cutoff standard.

#### Effect Analysis of Differential m6A Methylated Genes on the Related mRNA Expression Levels

Differentially expressed genes were recognized from microarray and TCGA and GTEx data by fold change and *P* value together analyzed with *t* test. Consequently, we performed a combined analysis of differently m6A methylated genes and differently expressed genes by the standard of *P* < 0.05 and | fold change| > 2.

#### Validation of m6A Modified Gene Expression Levels From Microarray by Matching TCGA and GTEx Data

To confirm the results, we validated the levels of m6A modified gene expression in stomach adenocarcinoma (STAD) by using the TCGA and GTEx data. | logFC| > 1 and *P* < 0.05 were considered to indicate statistically significant differences. A boxplot graph was produced for the visualization of the results.

#### Validation of m6A Modified Gene Expression Levels From Microarray in Human Tissues

We further verified the hub genes using 10 pairs of tumor and adjacent non-tumor tissues in GC that were matched according to age and sex (this validation was approved by the Human Ethics Review Committee of the First Hospital of China Medical University). Quantitative real-time polymerase chain reaction (PCR) was performed to profile the mRNA expression levels. Differences between the groups were compared by the use of a Mann–Whitney *U* test, and *P* < 0.05 was considered to be statistically significant. All of the primers that were used are shown in Table 1.

**TABLE 1:** Real-time PCR primer sequence.

Name		Sequence
SCD	F	CTTTCTGATCATTGCCAACACA
	R	TGTTTCTGAAAACTTGTGGTGG
MTHFD1L	F	GAGGAAGTGAGTAAATTTGCCC
	R	GCCCGATGGTTATTTTTCGTAG
IGF2	F	CTGGAGACGTACTGTGCTAC
	R	CATATTGGAAGAACTTGCCCAC
ORM1	F	TAACACCACCTACCTGAATGTC
	R	AAAAGCAAGCATGTAGGTCTTG
FGA	F	GGATCGTCTGCCTGGTCCTA
	R	CCTTCAGCTAGAAAGTCACCTTCA
GC	F	AAATGATGAAATCTGTGAGGCG
	R	AGCTTGTCCGTAATTAGTGGAA
GIF	F	GCCCTCTACCTCCTGAGCCTTC
	R	GCTGATGAAGTCACCGAGTTCTCC
MT1E	F	CATTCTGCTTTCCAACTGCCTG
	R	GCAGCTCTTCTTGCAGGAGG
GALNT1	F	ATGGCCCAGTTACAATGCTC
	R	ATATTTCTGGCAGGGTGACG
beta-actin	F	ATGTGGCCGAGGACTTTGATT
	R	AGTGGGGTGGCTTTTAGGATG

#### Clinical and Pathological Correlation and Survival Analysis of m6A Modified Genes by TCGA Data

We considered the differentially m6A modified genes as hub genes, and we performed clinical and pathological correlation and survival analyses of the TCGA data that were based on gene expression. A boxplot graph was produced for the visualization of the relationships. Overall survival was assessed by using Kaplan–Meier survival curves, and the hazard ratio with 95% confidence interval information and log-rank *P* values were calculated and included in the survival plots. Values of *P* < 0.05 were considered to indicate significant differences.

## Results

### Common Features of the m6A Modification of mRNA in GC and PCa Tissues

**FIGURE 1:**
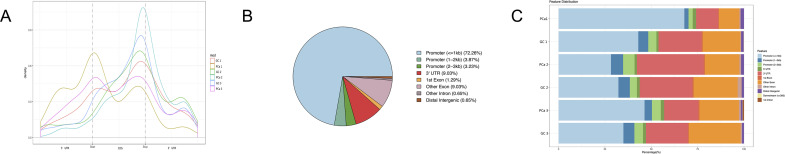
Distribution of m6A peaks along transcripts. **(A)** Each transcript is divided into three parts: 5’ untranslated regions (5’ UTRs), coding sequence (CDS), and 3′ untranslated region (3′ UTRs). **(B)** The m6A peak distribution within different gene contexts. **(C)** The m6A peak distribution along a metagene.

After removing low-quality data, approximately 9,700,000 to 21,200,000 high-quality reads were obtained from every PCa IP sample, as well as approximately 14,800,000 to 24,200,000 reads from every GC IP sample, and they were mapped to the reference genome with an efficiency of greater than 66%. Moreover, we found the consensus sequence GGACAR (*R* = U or A) C by analyzing the top 100 most significant peaks from every sample using MEME software (Machanick and Bailey, 2011); this sequence resembles the common m6A motif described in human diseases. Furthermore, the results showed that m6A peaks were significantly related to different gene locations in both GC and paired PCa tissues: near the middle of the 5’ UTRs, start of the coding sequence (CDS), near the end of the CDS, and 3′ UTRs. Moreover, for most samples, the peaks at the end of CDS were more pronounced than those at the start, and to further confirm the preferential locations of m6A on transcripts, we investigated the metagene profiles of m6A peaks (Figures 1A–C).

### DMGs Between GC and Paired PCa Tissues

**FIGURE 2:**
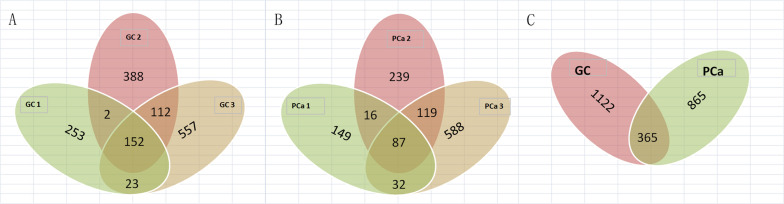
Statistics for genes with m6A modifications in each sample. **(A)** Number of genes with m6A sites in three GC samples; **(B)** number of genes with m6A sites in three PCa samples; **(C)** number of genes in GC and PCa tissues. We detected 1,487 m6A genes in GC tissues and 1,230 genes in PCa tissues, respectively, 365 genes were comethylated in GC and PCa tissues, accounting for 15.52% of the total methylated genes; 1,122 and 865 genes were only methylated in GC and PCa tissues, respectively.

**FIGURE 3:**
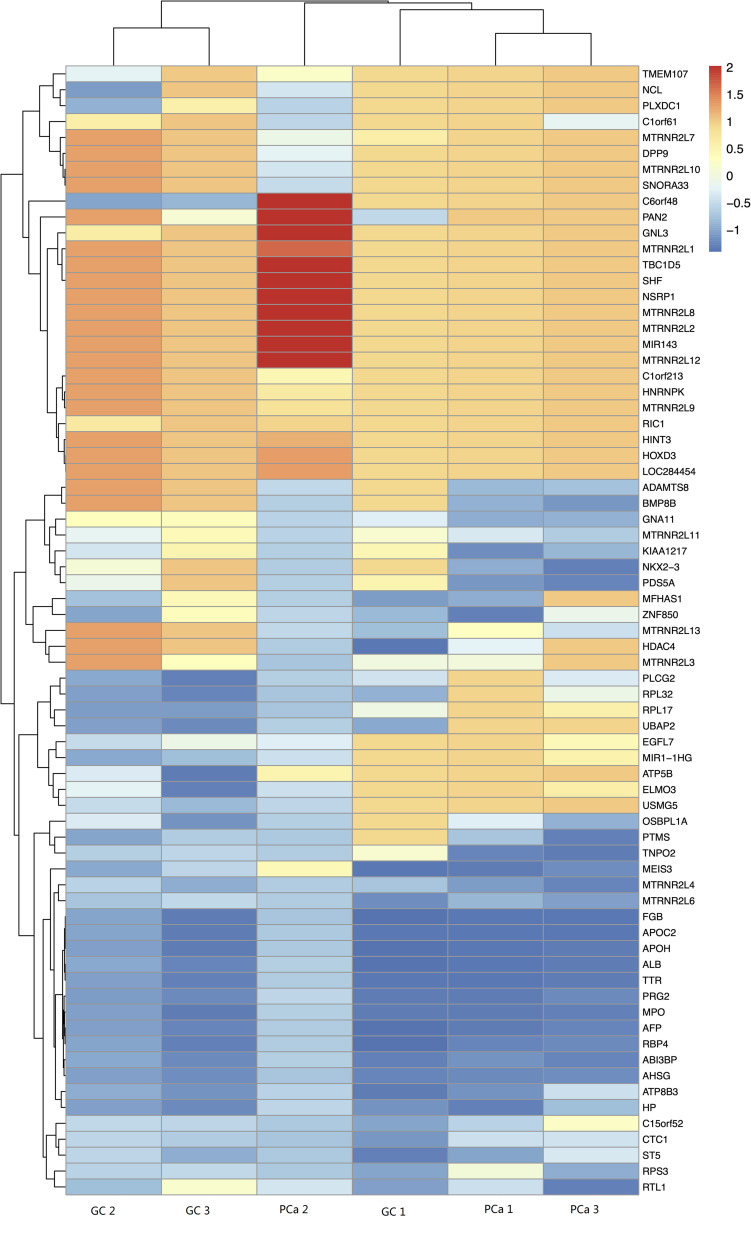
The heatmap of intersecting m6A signals for the represent group.

We detected 1,487 genes with 2,103 m6A sites in GC tissues and 1,230 genes with 1,688 m6A sites in PCa tissues. On average, 1.41 and 1.37 m6A sites occurred per gene mutation in GC and PCa tissues, respectively. A total of 365 genes were comethylated in GC and PCa tissues, accounting for 15.52% of the total methylated genes. A total of 1,122 and 865 genes were only methylated in GC and PCa tissues, respectively. Statistics for m6A genes of each sample are shown in a Venn diagram (Figure 2), and the heatmap of intersecting m6A signals for the represent group is shown in Figure 3. We searched for differential m6A modifications in mRNAs by analyzing GC and PCa tissues, and we identified 81 up-regulated and 62 down-regulated methylated protein coding genes (Supplementary Table S1). The top 10 up- and down-regulated genes and their related information are shown in Table 2.

**TABLE 2:** Top 10 up- and down-regulated differential methylated genes in GC tissues.

Catalog	Gene name	Chrom	txStart	txEnd	Peak ID	Fold change	*P* value
Up	F13A1	chr6	6152045	6152182	diffreps_peak_958	167	1.11E-16
	GOLPH3L	chr1	150620621	150620880	diffreps_peak_87	136	6.73E-14
	BDKRB2	chr14	96709521	96709720	diffreps_peak_355	131	1.78E-13
	BTNL9	chr5	180480468	180480620	diffreps_peak_947	114	0
	ZFHX3	chr16	72991561	72991820	diffreps_peak_492	91	1.02E-09
	PGP	chr16	2264561	2264822	diffreps_peak_449	81	8.92E-09
	PROC	chr2	128180681	128180984	diffreps_peak_677	70	0
	TLCD1	chr17	27053221	27053230	diffreps_peak_531	65	2.35E-07
	TSPAN9	chr12	3186520	3186560	diffreps_peak_253	64	0
	PANX2	chr22	50616361	50616740	diffreps_peak_794	57	0
Down	IST1	chr16	71929395	71929492	diffreps_peak_490	125	7.59E-13
	GIF	chr11	59612733	59612972	diffreps_peak_214	80	0
	PGA4	chr11	60989817	60989928	diffreps_peak_215	62	0
	PGA5	chr11	61008647	61008754	diffreps_peak_218	57	0
	SCOC	chr4	141264681	141264862	diffreps_peak_884	56	1.6E-06
	HES1	chr3	193853930	193854080	diffreps_peak_854	52	3.94E-06
	ATP5B	chr12	57038601	57038739	diffreps_peak_273	36	0
	PGC	chr6	41704448	41704580	diffreps_peak_1009	35	0
	LSS	chr21	47648401	47648738	diffreps_peak_776	31	5.17E-09
	RPL3	chr22	39715032	39715116	diffreps_peak_788	26	0

### DMGs Related Pathway and PPI Network Between GC and PCa Tissues

**FIGURE 4:**
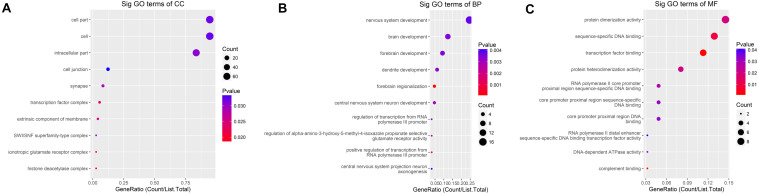
GO terms for mRNAs with increased m6A levels. **(A)** Cellular component, **(B)** biological process, **(C)** molecular function. The genes are mainly enriched in the histone deacetylase complex, ionotropic glutamate receptor complex, and transcription factor complex and are involved in a variety of molecular functions including transcription factor binding, complement binding, sequence-specific DNA binding, and biological progresses referred to forebrain regionalization and positive regulation of transcription from RNA polymerase III promoter.

GO analysis demonstrated that the up-methylated genes of m6A are chiefly enriched in the histone deacetylase complex, ionotropic glutamate receptor complex, and transcription factor complex. Further, these genes are engaged in various molecular functions, such as sequence-specific DNA binding, transcription factor–binding, and complement binding, and they are involved in biological progresses, such as forebrain regionalization and positive regulation of transcription from the RNA polymerase III promoter (Figure 4).

**FIGURE 5:**
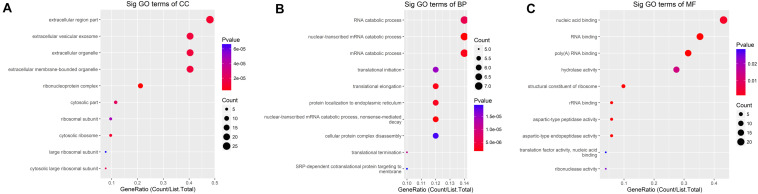
GO terms of mRNAs with decreased m6A levels. **(A)** Cellular component, **(B)** biological process, **(C)** molecular function. The genes are mainly enriched in the ribonucleoprotein complex, extracellular region part, and cytosolic ribosome and are involved in a variety of molecular functions including poly(A) RNA binding, RNA binding, aspartic-type endopeptidase and peptidase activity, and biological progresses referred to nuclear-transcribed mRNA catabolic process, translational elongation, and mRNA catabolic process.

The down-methylated genes of m6A modifications are mainly enriched in the ribonucleoprotein complex, extracellular region part, and cytosolic ribosome; further, the gene products are engaged in various molecular functions, such as poly(A) RNA binding, RNA binding, aspartic-type endopeptidase, and peptidase activity, and they are involved in biological progresses, such as nuclear-transcribed mRNA catabolic process, translational elongation, and mRNA catabolic process (Figure 5).

Furthermore, we investigated the Kyoto Encyclopedia of Genes and Genomes (KEGG) pathways of m6A methylated mRNAs. From the results, we found that the up-methylated mRNAs were mostly enriched in pathways including complement and coagulation cascades, transcriptional misregulation in cancer, and viral carcinogenesis. However, the down-methylated mRNAs were mostly involved in pathways including ribosome, vitamin digestion and absorption, antigen processing and presentation, protein digestion, and absorption.

**FIGURE 6:**
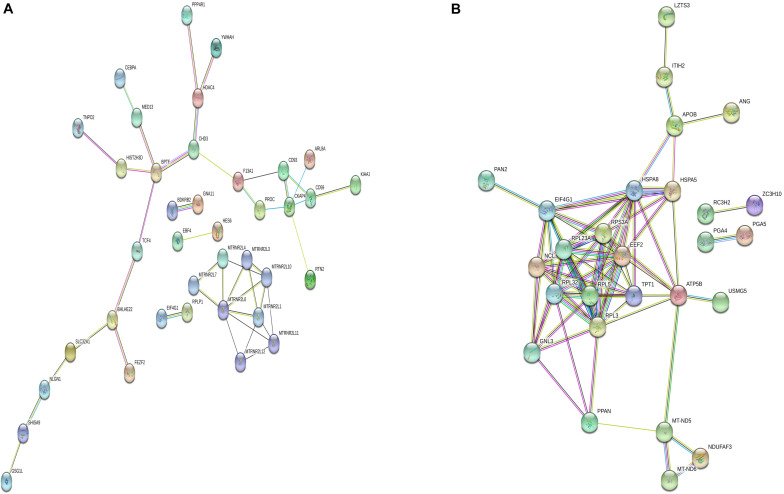
Protein–protein interaction network of DMGs. Disconnected nodes are not shown in the network. **(A)** total of 81 nodes and 44 edges were established in genes with increased methylation, and **(B)** 62 nodes and 74 edges were established in genes with decreased methylation. PPI networks both revealed a significantly more interactions.

Differentially m6A methylated genes were investigated using STRING. In total, 81 nodes and 44 edges were discovered in the up-regulated genes, and the PPI networks displayed even more interactions with a PPI enrichment *P* value of 9.08e-07 (Figure 6A). In addition, 62 nodes and 74 edges were discovered in down-regulated methylated genes, and the PPI networks displayed even more interactions with a PPI enrichment *P* value of 3.32e-12 (Figure 6B).

### The Effect Analysis of Differential m6A Modification of Genes on the Related mRNA Expression

**FIGURE 7:**
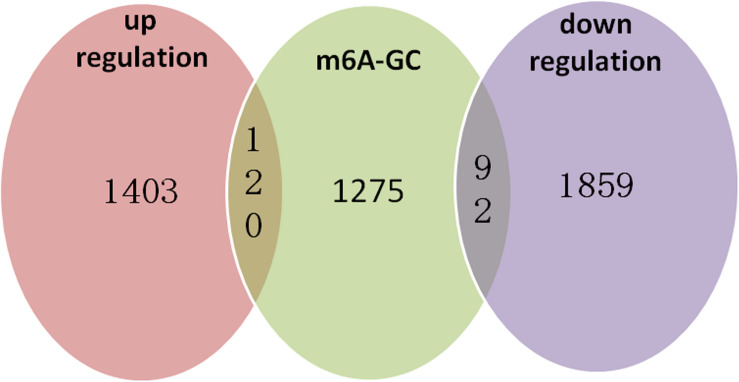
m6A methylation genes with the related mRNA differentially expressed genes in GC. We performed a combination analysis of 1,487 m6A methylation genes with related mRNA differently expression genes in GC and found 120 up-regulation genes and 92 down-regulation genes were related m6A methylation.

We identified 472 up-regulated genes and 346 down-regulated genes among three paired GC and PCa tissues, respectively (Supplementary Table S2). In addition, we analyzed 415 tumors and 262 normal control tissues in TCGA + GTEX database. A total of 3,017 differentially expressed genes were obtained, including 1,226 up-regulated genes and 1,791 down-regulated genes (Supplementary Table S3). We performed a combination analysis of 1,487 m6A methylation genes with the above mRNAs that were differentially expressed in GC. We found 120 up-regulated genes and 92 down-regulated genes related to m6A methylation (Figure 7). Further, we analyzed the differentially expressed genes related to differential m6A methylation. We finally identified 18 genes with increased m6A levels and 9 genes with decreased m6A levels that had differential m6A methylation between GC and PCa tissues. Among these genes, 15 genes revealed a positive correlation between m6A methylation and gene expression, whereas 12 genes had a negative correlation that may affect their expression in GC (Table 3).

**TABLE 3:** Gene and relationship between differential m6A methylation and gene expression levels.

Category	Up-methylated	Down-methylated
Hyperexpression	MTHFD1L	GALNT1
	IGF2	
	SCD	
	BMP8B	
	TSPAN9	
	TLCD1	
	CEBPA	
Hypoexpression	GC	
	ORM1	
	FGA	
	HS3ST4	MT1E
	SHISA9	GIF
	NLGN1	CPEB2
	BTNL9	PGA5
	PLD5	PGA4
	ADAMTS8	SLC13A3
	EBF4	RPL5
	F13A1	PGC

### The Differential Expression of m6A Modified Genes From Microarray Validated by Matching TCGA and GTEx Data and in Human Tissues

**FIGURE 8:**
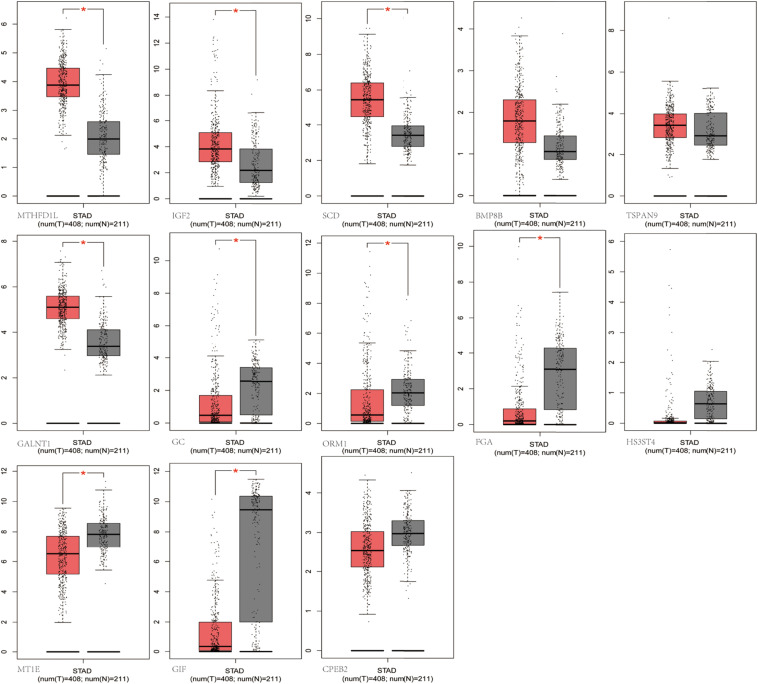
Validation of the differentially m6A related expressed genes. MTHFD1L, IGF2, SCD, GC, ORM1, FGA, GALNT1, MT1E, and GIF had significant differences (*P* < 0.05).

We selected the hub m6A modified genes from microarray to validate the expression in STAD by matching 408 tumor tissues and 211 normal tissues. The results demonstrated that nine hub genes, (MTHFD1L, IGF2, SCD, GC, ORM1, FGA, GALNT1, MT1E, and GIF) exhibited significant differences between the cancer and normal tissues. A boxplot graph was produced for the visualization of these differences (Figure 8). Next, we sought to verify the nine identified hub genes in human tissues and demonstrated that MT1E, GC, FGA, GALNT1, ORM1, and GIF exhibited statistical difference (*P* < 0.05); additionally, IGF2, MTHFD1L, and SCD exhibited no significant differences (*P* > 0.05), which may be related to the small sample size that we verified.

### Clinical and Pathological Correlation and Survival Analyses of Hub m6A Modified Genes by the Use of TCGA Data

**FIGURE 9:**
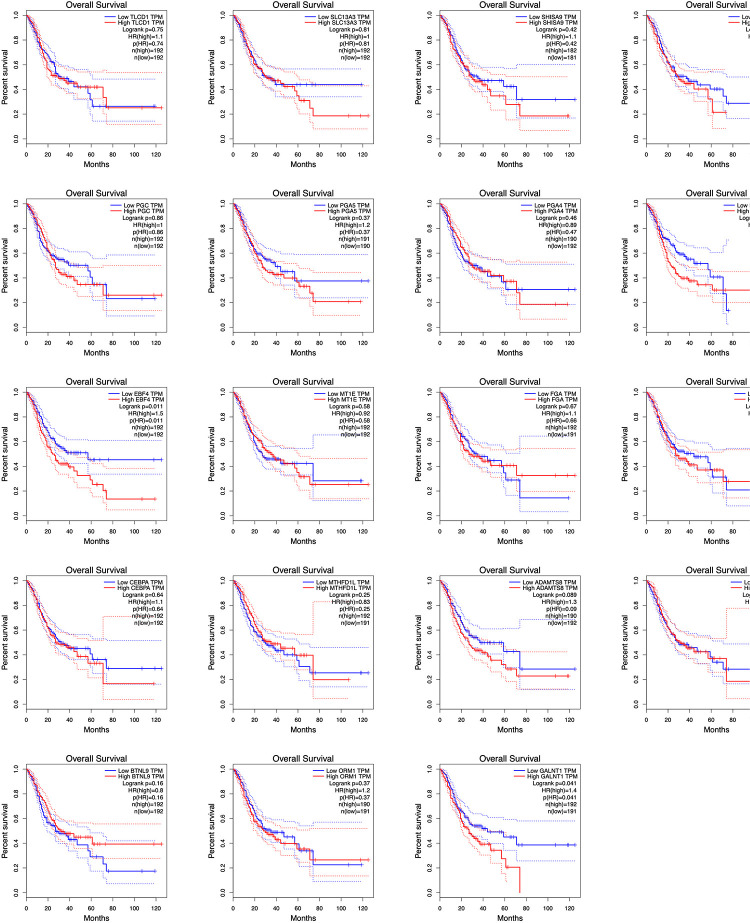
Survival analyses of hub genes by TCGA data. GNLAT1, EBF4, F13A1, and NLGN1 expression levels were related to the overall survival (*P* < 0.05).

Using TCGA data, we analyzed the correlation between the expression levels of hub m6A modified genes and clinicopathological parameters and prognoses. We observed that IGF2, SCD, GALNT1, TLCD1, SHISA9, NLGN1, and F13A1 expression levels were related to the T stage; IGF2, NLGN1, ADAMTS8, and F13A1 expression levels were related to the pathological stage; SCD, GALNT1, GIF, TLCD1, SHISA9, NLGN1, PLD5, ADAMTS8, F13A1, and SLC13A3 expression levels were related to the histological grade; ORM1, GIF, TLCD1, SHISA9, BTNL9, PGA5, PGA4, and RPL5 expression levels were related to anatomical subdivisions; ORM1 and F13A1 expression levels were consistently associated with metastasis; FGA expression levels were related to gender; NLGN1 expression levels were related to lymph node metastasis; PLD5 expression levels were related to reflux history; and EBF4 expression levels were related to Barrett esophagus (Supplementary Table S4). In addition, the survival analysis of the hub genes demonstrated that GNLAT1, EBF4, F13A1, and NLGN1 expression levels were related to the overall survival. The visualization of the survival analysis is displayed on the plots (Figure 9).

## Discussion

In this study, we first outlined the patterns and characteristics of m6A in GC, such as m6A modification of specific genes, distribution in transcripts, and the consensus m6A motif. In addition, we identified the DMGs between GC and PCa tissues and further analyzed their functions and enrichment pathways using bioinformatics tools. Finally, by combining analysis of differently m6A methylated and expression genes, we discovered some key genes that may exhibit dynamic expression because of m6A methylation and that are associated with GC risk and prognoses.

Previous studies have proven that the consensus motif sequence RRACH is the characterized in the m6A motif (Wei and Moss, 1977; Harper et al., 1990), and this has further been confirmed in many high-throughput m6A RNA sequencing databases (Dominissini et al., 2012; Luo et al., 2014). In our study, we found the consensus sequence GGACAR (*R* = U or A) C, which resembles the common m6A motif described in human diseases. The consistent sequence detected in the present study suggested that m6A methylation was conserved among various organs and tissues. Although, as mentioned before, m6A is mainly enriched in the vicinity of stop codons and 3′ UTRs in most mRNAs from different mammalian organs (Batista et al., 2014; Li et al., 2014), the distribution of m6A could be affected by the origin of tissue and genetic backgrounds of organisms, and the modified sites could remain unchanged, while the modified segments of each site may vary based on environmental factors (Luo et al., 2014). We found that m6A in both GC and PCa tissues was enriched around the CDS and at 3′ UTRs, as had been found in mammals. The m6A methylation peaks for most GC tissues were higher at the end of a CDS than they were at the start. According to our results, we speculate that when the stomach tissue evolves from a normal lesion to a cancerous lesion, changes in genetic and microenvironment factors are most focused on the end of the CDS. Moreover, the expansively abundant m6A modifications at the CDS or 3′ UTRs may be in charge of RNA stability, transport, and translocation signals or protein synthesis (Niu et al., 2013); these observations may also demonstrate a molecular mechanism, but further study is needed to verify these findings.

N6-methyladenosine RNA modifications show population-specific regulation at the cellular or species level in response to environmental changes (Luo et al., 2014; Tao et al., 2017). Some present studies have shown that m6A differs between cancers and adjacent tissues, such as HCC, cervical cancer, and breast cancer (Zhang et al., 2016b; Ma et al., 2017; Wang et al., 2017). Here, we detected an average of 1.41 and 1.37 m6A sites per gene mutation in GC and PCa tissues, respectively. In addition, we found that approximately 15.52% of genes were comethylated, but most of them were methylated separately in the GC and PCa tissues. In total, we identified 81 up-regulated and 62 down-regulated differentially methylated protein coding genes in GC, such as MTHFD1L, IGF2, SCD, BMP8B, TSPAN9, GC, ORM1, FGA, HS3ST4, GALNT1, MT1E, GIF, and CPEB2, suggesting that the epigenetic mechanism of mRNA may lead to the development of GC through m6A modifications.

Regarding the function and pathway of DMGs, we performed GO, KEGG enrichment analysis, and PPI construction for DMGs. The genes with increased m6A levels in their mRNAs are mainly enriched in transcriptional misregulation in the carcinogenesis pathway and are engaged in various transcription factor–binding functions. In accordance with previous observations, hypermethylation of transcription factors may give signal recognition and change the stability of the target transcripts related to carcinogenesis (Dominissini et al., 2012; Niu et al., 2013; Wang et al., 2014). The genes with fewer m6A modifications in their mRNAs mainly regulate ribosome, digestion and absorption of vitamin and protein, antigen processing, and presentation signal pathways; further, they are engaged in various molecular functions, such as RNA binding, aspartic-type endopeptidase, and peptidase activity. According to the PPI network produced by the identified DMGs, more interactions that were expected to be found were observed. These findings indicated that m6A methylation affected metabolic processes that reflected the specific functions and activities of the cancerous progression in GC.

The significance of m6A in many biological processes has mainly been studied in relation to the regulation of expression of m6A-related genes (Liu and Pan, 2016; Zhao et al., 2020). For the first time, we performed a combination analysis among the m6A methylated genes and their expression. Further validation of the differential expressed genes by matching TCGA normal and GTEx data, we identified six genes with increased methylation (MTHFD1L, IGF2, SCD, GC, ORM1, and FGA) and three genes with decreased methylation (GALNT1, MT1E, and GIF), which may be key m6A-related genes that play a role in gastric carcinogenesis as indicated by their regulated expression levels. We further performed quantitative real-time PCR to profile mRNA expression levels. The results showed that MT1E, GC, FGA, GALNT1, ORM1, and GIF had statistical difference (*P* < 0.05), and IGF2, MTHFD1L, and SCD had no statistical difference (*P* > 0.05). Previous studies discovered that one of the main functions of m6A is to mediate mRNA degradation (Liu et al., 2014; Wang et al., 2014), suggesting that there may be a negative correlation between the degree of m6A methylation and the level of transcription. However, in our results, four genes revealed a negative relationship between m6A methylation and gene expression, whereas five genes showed a positive correlation. According to our pathway analysis, increased m6A levels are mainly enriched during transcriptional misregulation in carcinogenesis, while decreased m6A mainly functions with digestion and absorption of protein. We speculate that m6A is related to the degradation of some genes in GC patients and is more likely to promote their protein translation and transcription factor–binding functions. Although these findings may play a significant guiding role in the progression of GC caused by m6A modification-related special genes, it needs to be confirmed by further studies on the mechanism and functional regions of these key genes.

In this study, we analyzed the correlation between the expression of m6A modified genes and clinicopathological parameters and prognoses. We demonstrated the hub genes (IGF2, SCD, GALNT1, TLCD1, SHISA9, NLGN1, F13A1, ADAMTS8, GIF, PLD5, SLC13A3, ORM1, BTNL9, PGA5, and PGA4, and RPL5, FGA, and EBF4) were related to clinical and pathological indices, including gender, anatomical subdivisions, reflux history and Barrett esophagus, histological grade, pathological stages, and TNM stages. In addition, GNLAT1, EBF4, F13A1, and NLGN1 expression levels were related to the overall survival. High expression levels were consistently associated with worse overall survival for 5-year survival rate, and the relative risk of death in patients with high expression levels was 1.4 or 1.5 times higher than that in patients with low expression levels. Briefly, the m6A related differently expressed genes may have potential predictive and prognostic values and can be used as m6A methylation-based biomarkers for precise GC diagnoses and treatments. However, a long-term follow-up of a large sample of clinical data is needed for further verification.

In conclusion, we first comprehensively analyzed the different m6A features of mRNA methylation between GC and paired PCa tissues and their potential impact on the related mRNA expression. We confirmed that the consensus m6A motif sequence was GGACAR (*R* = U or A) C and that the m6A peaks at the end of the CDS were more pronounced than they were at the start. Through the analysis of differential m6A methylated and expressed genes, we confirmed that m6A may play a role mainly through transcriptional misregulation in carcinogenesis or digestion and absorption of protein pathway, which then affects the expression of specific genes related to GC progression. The key genes IGF2, SCD, GALNT1, TLCD1, SHISA9, NLGN1, F13A1, ADAMTS8, GIF, PLD5, SLC13A3, ORM1, BTNL9, PGA5, and PGA4 and RPL5, FGA, and EBF4 were related to clinicopathological parameters and prognoses, which may be used as novel m6A methylation-based molecular markers that can provide accurate targets for the diagnosis and treatment of GC.

## Data Availability Statement

MeRIP-seq data has been uploaded to SRA repository (PRJNA665272). Microarray data has been uploaded to GEO repository (GSE158662).

## Ethics Statement

The studies involving human participants were reviewed and approved by the institutional ethics board of the First Hospital of China Medical University. The patients/participants provided their written informed consent to participate in this study.

## Author Contributions

YY contributed to study design and revising the manuscript. LS contributed to data interpretation and drafting manuscript. LPS and AW contributed to sample collection and data interpretation. HZ contributed to qPCR validation. All authors contributed to the article and approved the submitted version.

## Conflict of Interest

The authors declare that the research was conducted in the absence of any commercial or financial relationships that could be construed as a potential conflict of interest.

## Footnotes

1 http://string-db.org/
